# Effects of Geriatric Emergency Department Implementation on Hospital Admissions: Evidence from HCUP

**DOI:** 10.1093/geroni/igaf122.4107

**Published:** 2025-12-31

**Authors:** Jasmine Su, Cameron Gettel, Inessa Cohen, Xi Chen, Yuting Qian, Craig Rothenberg, Ula Hwang, Elyssa Grogan

**Affiliations:** New York University School of Medicine, New York, New York, United States; Yale School of Medicine, New Haven, Connecticut, United States; Yale University, Yale University, Connecticut, United States; Yale University, New Haven, Connecticut, United States; Yale School of Public Health, New Haven, Connecticut, United States; Yale University School of Medicine, New Haven, Connecticut, United States; NYU Grossman School of Medicine, New York, New York, United States; NYU Langone Health, New York, New York, United States

## Abstract

Since 2018, over 500 emergency departments (EDs) have been accredited as geriatric emergency departments (GEDs) in recognition of the age-friendly care they deliver to older adults. However, the impact of GED implementation on healthcare utilization remains unclear. This retrospective study examined the association between GED status and hospital admission using encounter-level data from the Healthcare Cost and Utilization Project (HCUP) from 2017-2022. 350 EDs, encompassing 20 million encounters, were matched 1:1 by GED status using hospital urbanicity, teaching status, state, encounter volume, and geriatric encounter volume. A difference-in-differences event-study design assessed the effect of GED status on hospital admissions over time in seven states. Analyses controlled for patient age, gender, ethnicity, and all matching variables above, with state, year, and ED fixed effects included. Event study estimates indicated a statistically significant decline in admissions at GED sites compared with matched non-GED sites. Relative to non-GEDs, GED implementation led to a 0.5-percentage-point decrease after one year (95% CI: −0.03, 0.02), a 4.6-point decrease (95% CI: −0.08, −0.01) after two years, a 7.5-point decrease (95% CI: −0.12, −0.03) after three years, and an 11.3-point decrease (95% CI: −0.17, −0.06) after four years, from a baseline mean of 51.6% in GED admissions. We saw no indication of pre-trends in the hospital admission outcome, indicating comparability between GEDs and non-GEDs prior to accreditation. These findings suggest that GED implementation may produce meaningful reductions in hospital admissions over time, supporting its potential to improve care for older adults in emergency settings.

